# The Transcription Factor PU.1 Regulates γδ T Cell Homeostasis

**DOI:** 10.1371/journal.pone.0022189

**Published:** 2011-07-14

**Authors:** Rukhsana Jabeen, Hua-Chen Chang, Ritobrata Goswami, Stephen L. Nutt, Mark H. Kaplan

**Affiliations:** 1 Department of Pediatrics, Herman B Wells Center for Pediatric Research, Indiana University School of Medicine, Indianapolis, Indiana, United States of America; 2 Department of Microbiology and Immunology, Indiana University School of Medicine, Indianapolis Indiana, United States of America; 3 The Walter and Eliza Hall Institute of Medical Research, Victoria, Australia; Oklahoma Medical Research Foundation, United States of America

## Abstract

**Background:**

T cell development results in the generation of both mature αβ and γδ T cells. While αβ T cells predominate in secondary lymphoid organs, γδ T cells are more abundant in mucosal tissues. PU.1, an Ets family transcription factor, also identified as the spleen focus forming virus proviral integration site-1 (*Sfpi1*) is essential for early stages of T cell development, but is down regulated during the DN T-cell stage.

**Methodology/Principal Findings:**

In this study, we show that in mice specifically lacking PU.1 in T cells using an *lck-Cre* transgene with a conditional *Sfpi1* allele (*Sfpi1* ^lck−/−^) there are increased numbers of γδ T cells in spleen, thymus and in the intestine when compared to wild-type mice. The increase in γδ T cell numbers in PU.1-deficient mice is consistent in γδ T cell subsets identified by TCR variable regions. PU.1-deficient γδ T cells demonstrate greater proliferation in vivo and in vitro.

**Conclusions/Significance:**

The increase of γδ T cell numbers in Lck-Cre deleter strains, where deletion occurs after PU.1 expression is diminished, as well as the observation that PU.1-deficient γδ T cells have greater proliferative responses than wild type cells, suggests that PU.1 effects are not developmental but rather at the level of homeostasis. Thus, our data shows that PU.1 has a negative influence on γδ T cell expansion.

## Introduction

T cells are divided into two populations based on their surface expression of αβ and γδ T cell receptors (TCR). γδ T cells function in immunosurveillance playing a significant role in innate immunity, autoimmunity and allergic responses [1]. γδ T cells constitute only a small proportion (1–5%) of the lymphocytes that circulate in the blood and peripheral organs of most adult animals however; they are more widely distributed within epidermal and mucosal tissues, such as the skin, intestine and reproductive tract comprising up to 50% of T cells. Both αβ and γδ T cells arise from common multipotent DN precursors in the thymus that can be further separated into four DN subsets based on CD44 and CD25 expression [2], [3]. Commitment to the T cell lineage is complete at the DN3 stage where the cells undergo extensive DNA rearrangements at the β, γ and δ TCR loci in order to express functional TCR chains and make a choice between two developmental programs, one generating αβ T cell subsets and one generating the distinct characteristics and functions of γδ T cells [4], [5].

The molecular events involved in γδ lineage commitment are poorly understood. Numerous signals impact on the γδ T cell lineage including, TCR signal strength, notch signaling, IL-7R signaling and the presence of DP thymocytes [6]–[10]. Expression of the transcription factor Sox13 promotes γδ T cell development while opposing αβ T cell differentiation. Mice deficient in Sox13 exhibit impaired γδ T cell development but not αβ T cells [11]. The expression of c-Jun is not required for, and may antagonize, γδ T cell development [12]. The role of other transcription factors in regulating γδ T cell development and peripheral homeostasis has not been examined.

PU.1 is an Ets family transcription factor that is essential for lymphoid and myeloid development [13]. PU.1 mRNA is expressed in hematopoietic stem cells and in the earliest thymic precursors but is downregulated during the pro-T cell stage. Decreased expression of PU.1 is necessary for continued progress through T cell differentiation [14]. Our lab has previously shown that αβ T cell development is normal in mice that conditionally lack PU.1 in T cells [15]. However the expression of PU.1 in T cells and subpopulations of Th2 cells contributes to heterogeneity in Th2 cytokine expression and TCR expression [16]. In this report we have shown that deletion of PU.1 results in increased numbers of γδ T cells in various organs. Our results suggest that PU.1 has negative influence on γδ T cell expansion in the periphery.

## Results

### γδ T cell numbers are expanded in the absence of PU.1

**Figure 1:**
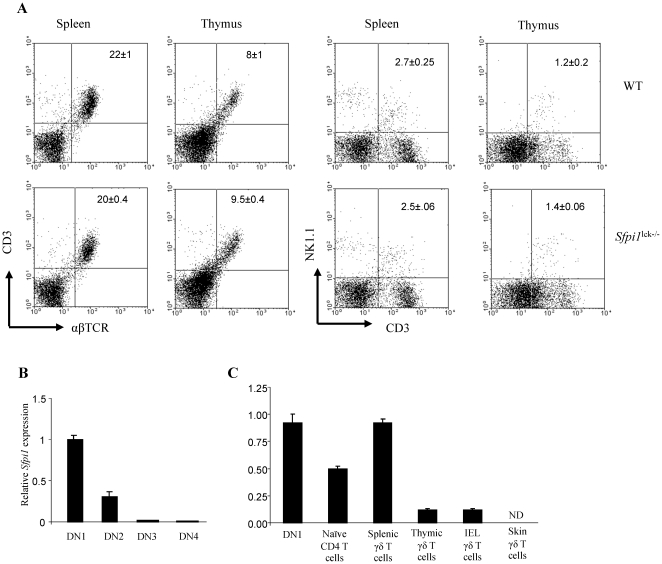
T cell development is normal in *Sfpi1* ^lck−/−^ mice. (A) Flow cytometric analysis of cells in spleen and thymus from *Sfpi1* ^lck−/−^ mice and wild-type control mice. The CD3+ αβTCR+ and CD3+ NK1.1+ T cell percentages are presented as mean ± SEM of six mice. Percentages were not significantly different between WT and *Sfpi1* ^lck−/−^ mice (p>0.05). (B) Thymocytes from wild-type control mice were sorted into double-negative subpopulations and RNA was analyzed for *Sfpi1* expression. (C) The indicated T cell populations were sorted and RNA was analyzed for *Sfpi1* expression by qPCR. Results are averages ± SEM of values from three mice. ND, not detected.

Previous work demonstrated that PU.1 functions in T cells by contributing to heterogeneity of TCR expression and Th2 cytokine production, and promoting the Th9 phenotype, while the development of αβ T cells was not affected in the absence of PU.1 [15]–[17]. In the characterization of mice that conditionally lack PU.1 following *Lck*-Cre mediated deletion of *Sfpi1*, we examined the effects of PU.1-deficiency on other T cell subsets. Flow cytometry of splenocytes and thymocytes showed similar profiles of CD3+ αβTCR and CD3+ NKT cells between wild-type and *Sfpi1* ^lck−/−^ mice (Fig. 1A). This is consistent with progressively diminished expression of *Sfpi1* throughout the stages of DN T cell development in the thymus (Fig. 1B). However, *Sfpi1* is expressed in peripheral CD4+ naïve αβ T cells (Fig. 1C). We examined other T cell subsets and observed that splenic γδ T cells had greater *Sfpi1* expression than αβ T cells (Fig. 1C). *Sfpi1* expression was lower in γδ T cells isolated from thymus, or intraepithelial lymphocytes, and was undetectable in γδ T cells isolated from skin (Fig. 1C). These results are consistent with a previous report that detected PU.1 expression in γδ T cells derived from fetal thymic organ culture [18].

**Figure 2:**
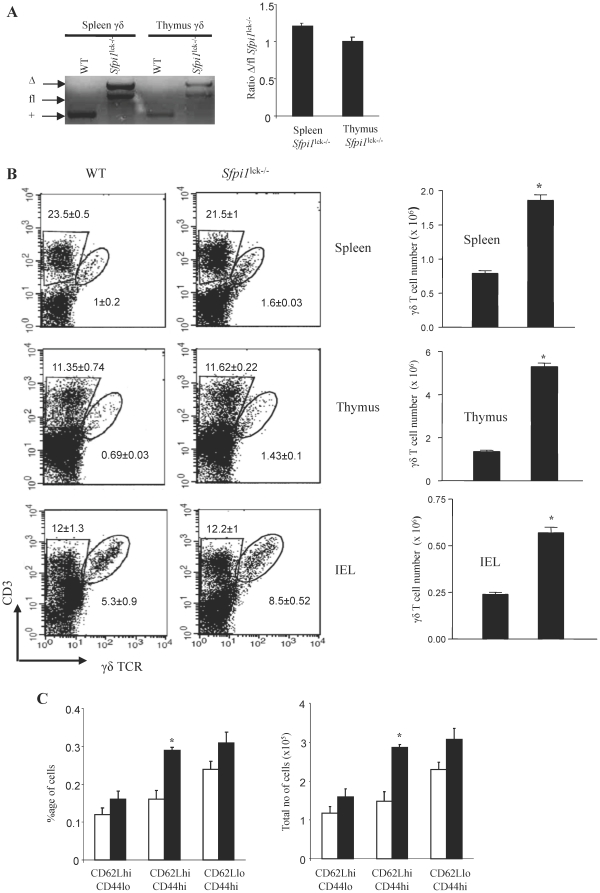
Increased γδ T cells in *Sfpi1* ^lck−/−^ mice. (A) γδ TCR+ thymocytes and γδTCR+ splenocytes were sorted from wild-type and *Sfpi1* ^lck−/−^ mice and genomic DNA was analyzed for the presence of the wild-type (WT; +), floxed (fl), or deleted (Δ) allele. (B) Flow cytometric analysis of γδ T cells in spleen, thymus and intestine (intra-epithelial lymphocytes) from wild-type and *Sfpi1* ^lck−/−^ mice using antibodies specific for CD3 and pan-γδ TCR. Numbers in dot plots represent the mean ± SEM of 10–12 mice. The absolute number of γδ T cells was calculated by multiplying the total cell number recovered from each organ by the percentage of γδ T cells. Results are an average of 10–12 mice. (C) Flow cytometric analysis of γδ T cells in spleen from wild-type and *Sfpi1* ^lck−/−^ mice using antibodies specific for pan-γδ TCR, CD62L and CD44. Numbers are γδ T cells in each subpopulation and are averages ± SEM of three mice. *Significantly different from WT, p<0.05 determined by Student's t test.

Thus we next examined γδ T cell in mice that lack expression of PU.1 in T cells. To determine if Lck-Cre deleted in γδ T cells, genomic DNA was isolated from purified γδ T cells from spleen and thymus and tested for deletion of the *Sfpi1* allele. Although deletion was not complete as previously observed in double-positive thymocytes [15], there was at least 50% deletion in γδ T cell populations (Fig. 2A). In contrast to the absence of an effect of PU.1 deletion on αβ T cells, we observed a consistent increase in the numbers of γδ T cells in a number of organs and tissues. Using flow cytometry to examine the percentages of cells positive for CD3 and γδ TCR, we observed significantly increased percentages of γδ T cells in spleen, thymus and among intra-epithelial lymphocytes (IEL) in the intestine, though not in the skin (Fig. 2B and data not shown). Since PU.1-deficiency does not significantly affect the overall cell number in these organs, the increased percentages of γδ T cells corresponds to increased numbers of γδ T cells in these organs (Fig. 2B). We further examined the expression of other surface proteins on wild type and PU.1-deficient splenic γδ T cells. Greater than 90% of the cells were CD5-positive regardless of PU.1 expression, and the increase in γδ T cells in the absence of PU.1 occurred similarly in CD24-positive and CD24-negative populations (data not shown). There were some differences in the expansion of γδ T cell subsets that expressed CD62L and/or CD44, and though all populations demonstrated a trend towards increased numbers in the absence of PU.1, only the CD62Lhi, CD44hi population was significantly increased (Fig. 2C).

### *Sfpi1* ^lck−/−^ mice contain enhanced numbers of γδ T cell subsets

**Figure 3:**
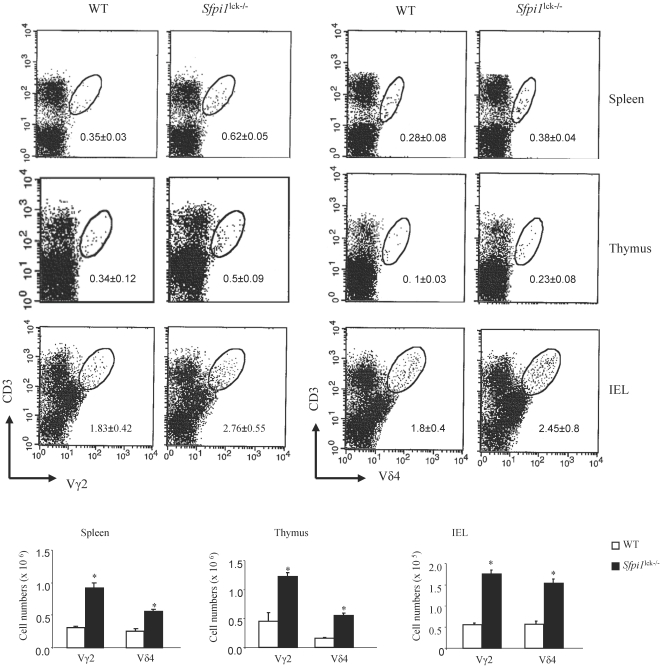
γδ T cell subsets in *Sfpi1* ^lck−/−^ mice. Flow cytometric analysis of γδ T cells in the indicated organs using antibodies specific for CD3 and individual Vγ or Vδ TCR regions. Numbers in dot plots represent the mean ± SEM of 4 mice and are representative of three independent experiments. The absolute number of individual γδ T cell subsets was calculated by multiplying the total cell number recovered from each organ by the percentage of γδ T cells. *, Significantly different from WT, p<0.05 determined by Student's t test.

To determine if these expanded populations included all subsets of γδ T cells identified by specific Vγ and Vδ TCRs, we examined the populations of cells that were positive for CD3 and Vγ2 or Vδ4. We observed significantly increased percentages of Vγ2+ and Vδ4+ T cells in all organs examined (Fig. 3). Concomitant with the increase in percentages, there was an increase in cell numbers of each of these γδ T cell subsets (Fig. 3).

### The absence of PU.1 results in enhanced γδ T cells proliferation

**Figure 4:**
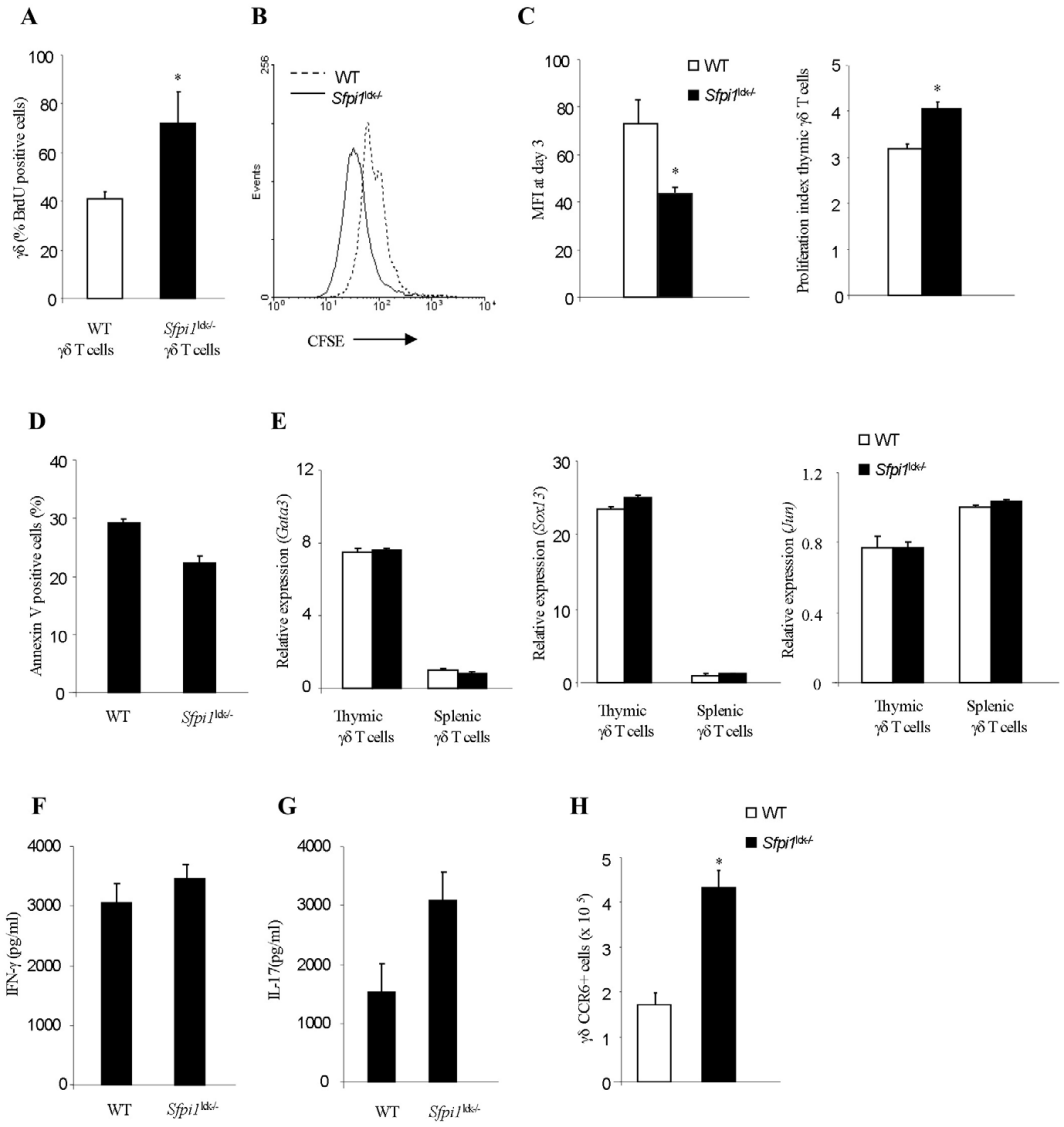
Proliferation and cytokine secretion from wild-type and Sfpi1^lck−/−^ γδ T cells. (A) Wild-type and *Sfpi1* ^lck−/−^ mice were injected with BrdU and after 24 hours thymocytes were stained with antibodies to γδ TCR and BrdU. Results are average ± SEM. (B–C) Splenocytes were stained with CFSE and stimulated with anti-CD3 and anti-CD28, and analyzed for the CFSE staining on γδ T cells after 72 hours. CFSE staining profiles are shown (B). The mean fluorescence intensity of CFSE and the proliferation index of the populations are presented as the mean ± SEM of three mice. (D) γδ T cells from spleen of wild-type and *Sfpi1* ^lck−/−^ mice were stained with PE-Annexin V and percentages of annexin V positive cells are indicated. (E) RNA was isolated from sorted γδ T cells from spleen and thymus of wild-type and *Sfpi1* ^lck−/−^ mice before analysis of mRNA using qPCR. Results are the average ± SEM of 5–6 mice. (F-G) γδ T cells were purified from spleen by flow cytometry and stimulated with anti-CD3 for 3 days before IFN-γ and IL-17 concentrations in supernatants were quantified using ELISA. Data are representative of two independent experiments and represent mean ± SEM of replicate samples. (H) γδ T cells in spleen from wild-type and *Sfpi1* ^lck−/−^ mice were stained using antibody specific for CCR6. Numbers are γδ T cells in each subpopulation and are averages ± SEM of three mice. *, Significantly different from the WT, p<0.05 determined by Student's t test.

We next examined whether PU.1-deficiency affected γδ T cell function or gene expression. The expansion of γδ T cells in the absence of PU.1 suggested that cell proliferation might be affected. To assess this in vivo we injected mice with BrdU to determine the percentage of γδ T cells in cell cycle. We observed a significant increase in the percentage of BrdU-positive PU.1-deficient γδ T cells, compared to wild type cells (Fig. 4A). To define proliferation in vitro, thymic cells were labeled with the fluorescent dye CFSE and activated with plate bound anti-CD3 and anti-CD28 for 72 h. Cell division was analyzed by flow cytometry. γδ T cells from *Sfpi1* ^lck−/−^ mice had greater proliferation than cells from wild-type mice, determined by staining profile, mean fluorescence intensity of CFSE staining, and proliferative index (Fig. 4B–C). In contrast, PU.1 had only modest effects on apoptosis (Fig. 4D). Using qPCR we tested the expression of transcription factors previously shown to affect γδ T cell development, including *Gata3*, *Sox13* and *Jun* [11], [12], [19]. We did not observe any significant differences in the expression of these transcription factors in γδ T cells from wild-type and *Sfpi1* ^lck−/−^ mice (Fig. 4E). Previous reports have demonstrated that γδ T cells can secrete IFNγ and IL-17 in response to TCR stimulation [20]–[22] and the cytokine secretion pattern may vary with antigen exposure [23]. To test the cytokine production from PU.1-deficient γδ T cells, we purified γδ T cells from wild-type and *Sfpi1* ^lck−/−^ splenocytes using flow cytometry and stimulated them with anti-CD3. After 72 hours, supernatants were collected and tested for amounts of IFNγ and IL-17 using ELISA. Amounts of IFNγ produced were similar between wild-type and PU.1-deficient γδ T cells, consistent with similar expression of Tbx21 and with similar percentages of γδ T cells that were CD122-positive (Fig. 4F and data not shown). There was a trend towards more IL-17 production from PU.1-deficient γδ T cells than from wild type cells, though this was not statistically significant (Fig. 4G). Although there was no difference in the expression of *Rorc* between in wild-type and PU.1-deficient γδ T cells, we did observe a significant increase in the numbers of γδ T cells that were CCR6+, supporting a selective increase in the populations of some cytokine secreting γδ T cells in the absence of PU.1 (Fig. 4H and data not shown).

## Discussion

The development of γδ T cells is still not completely understood. Development is regulated by extracellular signals and the expression of cell-intrinsic factors that regulate development and expansion. In this report we identify PU.1 as a transcription factor that regulates numbers of γδ T cells in secondary lymphoid organs and at mucosal sites. This represents a distinct function of PU.1 between αβ and γδ T cells, since αβ T cell development and numbers in various lymphoid organs is unaltered by PU.1-deficiency. Thus, this study has identified a novel function of PU.1 and contributes further to our understanding of transcription factor control of γδ T cell homeostasis.

PU.1 is expressed in γδ T cells from several organs. Splenic γδ T cells have higher expression of *Sfpi1* than naïve CD4+ T cells, although *Sfpi1* is expressed in lower amounts by thymic and intraepithelial intestinal γδ T cells, and not expressed in dermal γδ T cells. Since dermal γδ T cells have a fetal thymic origin [1], it is possible that there may be a difference in PU.1 expression between γδ T cells that derive during embryonic development and mature mice. PU.1 expression was observed in γδ T cells derived from fetal thymic organ culture suggesting that a fetal thymus does not result in the absence of PU.1 expression in γδ T cells [18]. Further studies will be required to determine precisely when *Sfpi1* is regulated during γδ T cell development.

The point at which PU.1-deficiency affects γδ T cell homeostasis is difficult to define. Although γδ T cell commitment during DN T cell development may occur at several stages [24] deletion by Lck-Cre, which occurs after *Sfpi1* expression is diminished between DN1 and DN2 and extinguished by DN3, is unlikely to affect γδ T cell development. In support of this idea, we did not see alterations in the expression of transcription factors that mediate αβ/γδ T cell lineage decisions. Moreover, it is likely that the phenotype observed is cell-intrinsic, and not due to the reported effects of αβ T cells on γδ T cell development [10] because deletion of *Sfpi1* by CD4-Cre, where *Sfpi1* is deleted in all αβ T cells did not effect γδ T cell numbers in spleen or thymus (data not shown). We did observe that proliferation of thymic γδ T cells was increased in vivo and in vitro in the absence of PU.1. However, this does not distinguish between proliferation of γδ T cells during or after development, and it is still possible that PU.1 functions at both stages. The precise functions PU.1 regulates will require a greater understanding of γδ T cell homeostasis.

We demonstrate that PU.1-deficiency increases in vivo proliferation of γδ T cells, and in vitro TCR-induced proliferation of γδ T cells. In several cell types PU.1 can function as a tumor suppressor [25], [26]. The development of myeloid leukemia in mice that have decreased expression of PU.1 is dependent on Jun [27]. Thus while Jun is not required for γδ T cell development [12], and we did not observe differences in the expression of *Jun*, it is possible that in the absence of PU.1, Jun may help to promote expansion of T cells. In various cell types PU.1 has also been shown to induce expression of TRAIL and Ink4b, which respectively induce apoptosis and inhibit cell proliferation [28], [29]. It may ultimately be a combination of effects on multiple genes that allows PU.1 to regulate γδ T cell expansion.

In the initial descriptions of PU.1, it was thought to be largely restricted to myeloid lineages [30]. During T cell development PU.1 expression is decreased between DN1 and DN2, and is extinguished by DN3. However, PU.1 is expressed in naïve CD4+ T cells, and we have shown that it regulates the expression of TCR in T cells, decreasing the threshold of activation [15]. We have also demonstrated that PU.1 limits the expression of Th2 cytokines and contributes to Th2 heterogeneity [15], [16]. Importantly, PU.1 promotes the development of Th9 cells [17]. In contrast to these effects, we did not observe significant effects of PU.1-deficiency on cytokine production from γδ T cells. Thus, PU.1 appears to have distinct effects in various types of T cells, negatively regulating γδ T cell numbers, negatively regulating Th2 cytokine production and TCR expression, while promoting IL-9 expression [15]–[17].

In this report we describe the effects of PU.1-deficiency on the γδ T cell population. The transcription factor network that promotes the development and homeostasis of γδ T cells is not well-defined. Our demonstration that deletion of PU.1 results in expansion of γδ T cells, an effect that is restricted to this subset, adds to our understanding of the regulation of γδ T cells. Further work will help to define how this factor limits γδ T cell expansion.

## Materials and Methods

### Ethics Statement

Mice were maintained in pathogen-free conditions and all studies were approved by the Indiana University School of Medicine Animal Care and Use committee.

### Mice

Wild-type C57BL/6 female mice were purchased from Harlan Bioscience. Mice with conditional deletion of the PU.1 gene (*Sfpi1* ^lck−/−^) on the C57BL/6 background were previously described [31] and mated to mice carrying a Cre transgene under control of an *Lck* promoter (B6(CBA)-Tg(Lck-cre)I540Jxm/J). The examination of allele deletion was performed as previously described [31]. Mice were used at age 6–8 weeks.

### Cell Isolation and Flow cytometry

Splenocytes and thymocytes of both wild-type and *Sfpi1* ^lck−/−^ were harvested and single cell suspensions were obtained. Viable cells were counted and determined by trypan blue exclusion. IELs were isolated by incubation of cleaned intenstine followed by Ficoll gradient centrifugation as described previously [32]. Splenocytes, thymocytes and IELs were then preincubated with Fc-block (2.4G2, BD pharmingenTM) for 10 minutes, followed by incubation with anti-CD3 PE, anti-γδTCR FITC, anti-Vγ2δTCR FITC, anti-Vδ4TCR FITC or antibodies to other surface proteins for 30 minutes at 4°C. Stained cells were analyzed with the BD FACSCalibur flow cytometer. All antibodies were purchased from BD Pharmingen unless otherwise stated.

### Cell proliferation and apoptosis assay

For BrdU studies, mice were administered 2 mg of BrdU intra peritoneally 24 h prior to analysis. To detect incorporation of BrdU, cells were stained with mAb specific to BrdU using a FITC-BrdU flow kit (BD Biosciences) following manufacturer's instructions.

For CFSE labeling, γδ T cells were isolated from both wild-type and *Sfpi1* ^lck−/−^ by staining splenocytes and thymocytes with PE anti-γδTCR before sorting with FACSAria (Becton Dickinson). Sorted γδ T cells from spleen of wild-type and *Sfpi1* ^lck−/−^ mice were washed in PBS, resuspended at a concentration of 10^6^ cells/ml and incubated with 5 µM of CFSE (Invitrogen) at 37°C for 10 minutes. Cells were then washed with cold culture media supplemented with 10% FBS. CFSE labeled cells were cultured for 3 days in anti-CD3 coated plates and soluble anti-CD28 (anti-CD3 4 µg/ml). The cell division status of cells was determined by measuring CFSE fluorescence after 3 days. Apoptosis was measured by annexin V staining according to the manufacturer's protocol (BD Pharmingen). Briefly, γδ T cells were washed with PBS supplemented with 10% FBS and then resuspended at a concentration of 10^6^ cells/ml in binding buffer. Cells were then stained with PE-Annexin V and incubated for 15 minutes. After incubation cells were resuspended in 400 µl of binding buffer and analyzed by flow cytometry within 1 h.

### Cytokine secretion assays

The purified γδ T cells were stimulated in vitro at 1×10^6^ cells/well in duplicate with 2 µg/mL plate-coated anti-CD3 antibody (BD Biosciences, San Jose, CA) for 72 hours. Supernatants were collected and assayed for the presence of cytokines IFN-γ and IL-17 by ELISA.

### Genomic DNA extraction and polymerase chain reaction analysis

The DNA was extracted from γδ T cells sorted from spleen or thymus using DNeasy Blood and Tissue kit from Qiagen. PCR was performed using primer/probe sets as described previously [31]. RNA was extracted from sorted γδ T cells from the indicated organs using Trizol. Quantitative real-time polymerase chain reaction was performed using Taqman Fast Universal PCR Master Mix and the plate was run on a 7500 Fast Real-Time PCR system (Applied Biosystems, Foster City, CA). RNA was normalized to expression levels of β2-microglobulin and relative expression was calculated using the -ΔΔCt method.
